# The Treatment of Colles's Fracture

**Published:** 1892-11-26

**Authors:** 


### ST. BARTHOLOMEW'S HOSPITAL.

### The Treatment of Colles's Fracture.

It is necessary, in the first place, to note that the treatment of the fracture we are discussing is almost invariably carried out by the house surgeon in the surgery, and is never, unless complicated by other and more serious injuries, seen by one of the surgeons to the hospital. The house surgeon, however, of course carries out the treatment as taught in the medical school.

There are three things to be aimed at in the treatment of this fracture, viz.: 1. Reduction of the deformity. 2. Maintenance when reduced in a fixed position. 3. After treatment for the prevention or cure of subsequent stiffness.

1. This is almost always accomplished at St. Bartholomew's, by extension on the hand, while counter-extension is being made by an assistant, who grasps the arm and forearm of the patient about the elbow. The amount of force necessary varies greatly in different cases, according to the presence or absence of much impaction. It is generally found, also, that the extension is much more efficient, if it tends somewhat towards the ulnar side, so as to overcome the displacement towards the radial side, it may also be much assisted by manipulation of the lower fragment with the left hand, especially if the impaction be not very firm.

2. In St. Bartholomew's the splints used are almost exclusively two simple straight splints, one placed on the flexor, and the other on the extensor aspect of the forearm. These splints are always used of such a width, that when the bandage is applied none of it comes in contact with the arm; by this means, too tight compression of the limb, with its attendant evils of pain, œdema, and possibly gangrene or sloughing is prevented.

The necessary width of splint is usually from three to four inches, according to the size of the arm.

A word may be here said on the method of padding splints at St. Bartholomew's. Pads should be firm, and at the same time somewhat yielding and elastic, and these conditions are found to be well fulfilled by the use of tow, well teazed out in a longitudinal direction, parallel with the length of the splint, and laid in several layers, until a fair thickness has been obtained. It is then covered with such material as old sheeting, which is firmly stitched to the back of the splint. It is found that tow, while cheaper, is also better than cotton wool, which latter is very liable to become lumpy in use.

Now as to the exact method of applying these splints. It must never be forgotten that the best position for the forearm in a case of fracture is that midway between pronation and supination; because in this position the radius and ulnar are farther apart from each other, and so there is less danger of ankylosis between them, which, if it took place, would mar the usefulness of the hand by crippling its movements.

In order to apply the splints in this position the patient is seated, and the elbow raised until it is on the same level as the shoulders; the palmar surface of the hand is then placed parallel with the floor (that is, the thumb is placed pointing towards the face of the patient). The splints, which should be long enough to reach from the elbow almost to the tips of the fingers, are then applied by an assistant, and firmly fixed by two pieces of strapping, while the reduced position of the fracture is maintained by the surgeon. A bandage is then applied over all, figure-of-eight is the method most usually adopted. All that is now necessary is to apply a sling to support the whole length of the forearm by means of a triangular bandage; the arm, when lowered to the chest, being found to lie comfortably with the flexor aspect against it. Another method is sometimes employed at St. Bartholomew's, Carr's apparatus being used instead of two straight splints. This consists of a splint for the flexor aspect, somewhat thinned at the wrist in order to fit it, and having obliquely fixed to its end a piece of ruler sufficiently long to be conveniently grasped in the hand. There is also a small straight splint for the extensor aspect. It is applied in the following way: Reduction is effected in the usual manner, and then the hand is made to grasp the palmar piece, and fixed by a turn or two of bandage. The extensor splint is then applied, and the whole firmly bandaged and put in a sling. Before application the splints are padded with a double layer of lint. The object of this splint is to overcome the displacement towards the radial side, and also for convenience in getting early movement of the fingers, and so to prevent stiffness.

Before going on to the subsequent treatment, a practical point may here be mentioned; namely, the importance of getting very good position in these fractures, since, if deformity is left, it not infrequently causes considerable interference with the utility of the hand. Even at St. Bartholomew's, with plenty of assistance obtainable, it is sometimes found necessary to give an anæsthetic for this purpose.

3. Stiffness of the fingers is a difficulty that has to be continually thought of in treating this fracture. It is usual at St. Bartholomew's in using the simple straight splints, to examine the condition of the bones at about the end of five days to a week, in order to see that they are in good position, and this opportunity is also generally taken of shortening the splint on the flexor aspect, so that it reaches only to the metacarpo phalangeal joints in order that passive movement of the fingers may be begun.

At the end of another fortnight the extensor splint may also be shortened, and passive movement tried at the wrist joint if there seems to be some firmness in the fracture. If Carr's splint has been used it is usual to free the fingers from their bandage at about the end of a week. All apparatus is generally given up at about the end of the fourth week, the arm being then simply enclosed in a bandage, and kept in a sling for about another week. A good deal of stiffness is usually left, and this is treated by friction, in order to facilitate which some form of liniment is given, such as the Linimentum Saponis of the Pharmacopœia. By several of the surgeons the treatment of the remaining stiffness is thought to be best carried out by bathing the arm and hand in water as hot as can be conveniently borne, then in the bath carrying out forcible passive movement as much as the patient will allow. After the bath the limb is thoroughly rubbed with the liniment. Should the stiffness not yield to this treatment, it has in some cases been advised to give an anæsthetic, such as nitrous oxide gas, and break down the adhesions which are giving most trouble by force, and subsequently carrying on the treatment by douches and frictions.

